# Operationalizing language-based population stratification for widening access to precision genomics in Africa

**DOI:** 10.3389/fpubh.2025.1672038

**Published:** 2025-09-12

**Authors:** Benard W. Kulohoma, Colette S. A. Wesonga

**Affiliations:** Ortholog, Nairobi, Kenya

**Keywords:** precision genomics, Africa, lexical similarity, multi-ethnic, population stratification, genomic

## Abstract

**Background:**

Despite remarkable advancements in genomic technologies, individuals of predominant African-related genetic similarity remain significantly under-represented, accounting for only 2.4% of published genome-wide association studies. This disparity limits our understanding of human biology and hinders equitable translation of genomic advances into healthcare.

**Methods:**

We exploited a quantitative framework using normalized Levenshtein distance (LDN) to analyse lexical similarity patterns across Kenya’s ethnolinguistic landscape, comprising Bantu, Nilotic, and Cushitic language groups. We compared lexical distance matrices with available genetic population differentiation data and geographic proximity to evaluate their relative efficacy in predicting genetic relationships.

**Results:**

Lexical similarity analysis revealed distinct clustering patterns that closely mirror Kenya’s ethnolinguistic diversity. Multidimensional scaling and hierarchical clustering clearly separated the three major language families and identified fine-scale relationships within each group. Importantly, lexical distance demonstrated stronger correlation with genetic differentiation [*r* = 0.91, CI (0.55–0.99)] than geographic proximity [*r* = 0.29, CI (0.29–0.53)], confirming language as a superior proxy for population genetic structure. Our analysis, demonstrate an objective basis for prioritizing populations in genomic studies.

**Conclusion:**

This study establishes lexical similarity analysis as a powerful alternative approach for predicting genetic relationships among diverse African populations. By enabling strategic prioritization of representative populations for genomic sequencing initiatives, this approach offers a practical solution to address the critical under-representation of African genetic diversity in global databases, with potential applications across Africa’s over 3,000 ethnic groups. This methodology provides a systematic, data-driven alternative to convenience sampling in regions where genetic data remains limited.

## Introduction

The landscape of modern genomics has been transformed by remarkable advancements in sequence data generation and analysis techniques. However, a fundamental challenge persists: the significant underrepresentation of diverse ancestral backgrounds in genetic studies. This disparity is particularly pronounced among individuals of predominant African-related genetic similarity who account for only 2.4% of published genome-wide association study (GWAS) data catalogued to date (1). The inclusion of these populations would undoubtedly enhance our understanding of human biology, potentially leading to novel drug discovery opportunities and clinical care benefits that extend far beyond these specific genetically similar groups identified from the 1,000 Genomes project (2, 3).

Africa’s population is characterized by extraordinary ethnic diversity, comprising over 3,000 genetically distinctive ethnic groups with significantly less linkage disequilibrium (LD) among loci compared to non-African populations (4). This genetic landscape presents a substantial challenge regarding how to prioritize representative populations for genomic sequencing initiatives. The genetic adaptations observed across these populations have evolved in response to diverse environmental pressures, including varied climates, diets, exposure to infectious diseases, and other factors that shape phenotypic adaptation.

These ethnic groups also exhibit significant variation in language and culture, characteristics that have been successfully leveraged alongside available genetic data to develop methodological frameworks for distinguishing populations and revealing historical migration patterns (5–9). Incongruence between genetic distance and lexical similarity could arise due to language shifts, gene flow, and recent admixture (10, 11). Linguistic patterns are thought to correlate more strongly with genetic structure than geographic proximity (9), particularly in African and Asian populations where coevolutionary patterns have been documented (8, 12). These findings suggest that lexical similarity analysis offers a powerful framework for identifying and prioritizing populations to generate more representative human genetic data.

Kenya, an East African nation with a population of 52 million, comprises 42 distinct ethnic groups that constitute a genetic tapestry shaped by separate migrations and adaptations. A small number Kenyan populations (n ~ 6) have already been represented in major human genetics initiatives, including the Luhya (LWK) in the HapMap and 1,000 Genomes projects, the Human Heredity and Health in Africa (H3Africa) project, the African Genome Variation Project (AGVP), and various published studies (2, 5, 13–15). However, these handful of people under-represents the diversity present in Kenya (*n* = 42 ethnic groups), and the wider African continent (> 3,000 distinct ethnic groups). Kenya’s population is distributed across three major language groups (Supplementary Table 1): Bantu, Nilotic, and Cushitic speakers, each with distinct historic migration routes into Kenya and sociocultural practices (Table 1). Here we test whether lexical similarity can serve as a predictive framework for genetic relatedness among diverse African populations. We demonstrate that linguistic patterns outperform geographic proximity in predicting genetic similarity, enabling strategic prioritization of population sampling to maximize the genetic diversity captured with minimal redundancy. Our quantitative lexical-based framework systematically identifies representative populations for genomic studies, accelerating the prioritization of underrepresented self-identified Africans with genetic similarity to those in 1000 Genomes panel samples for inclusion in global genetic databases. This provides an operationalizable precision health strategy for population-level genomic inclusion. This data-driven approach for stratifying diverse populations for inclusion in genomic studies is useful and scalable in resource-limited settings, with diverse ethnic populations, and fosters global health equity.

**Table 1:** The major Kenyan language groups and their demographic history.

Language group	Key ethnic groups	Migration route	Demographic history
Bantu (Niger-Congo)	Kikuyu, Kamba, Luhya, Kisii, Swahili	From West-Central Africa → across Central Africa → into Kenya, Tanzania, Uganda	Bantu Expansion (~2,000–3,000 years ago); farming communities moving eastward
Nilotic (Nilo-Saharan)	Luo, Kalenjin (Kipsigis, Nandi), Maasai, Turkana, Teso	From Nile Valley/South Sudan → into western Kenya, northern Uganda	Pastoralist migrations southward; settled near water bodies and highlands
Cushitic (Afro-Asiatic)	Somali, Rendille, Gabra, Oromo	From Horn of Africa → into northern and eastern Kenya	Older Afro-Asiatic presence; long-term contact with Nilotic and Bantu groups; trade and cultural exchange

## Methods

### Lexical distance estimation and visualization

Lexical similarity among the languages was assessed using normalized Levenshtein distance (LDN), applied to a standardized wordlist (Supplementary material) (16). LDN provides a transparent, interpretable measure for decision-makers, and is adaptable to multilingual, multi-ethnic contexts across Africa where genetic sequencing capabilities are constrained. LDN when averaged across aligned wordlists, reliably estimates lexical distance between languages and enables the construction of language phylogenies (17). We calculated pairwise LDN across all word pairs sharing the same translation in the wordlists. These were then averaged per language pair to generate a distance matrix. Using this matrix, we performed multidimensional scaling (MDS) to project the distances into two-dimensional space and constructed a hierarchical clustering dendrogram using Ward’s method (16, 17). A heatmap of lexical distances was also generated for comparative visualization. All analyses and visualization were conducted in R (18). Our analyses scripts are open-source and can be adapted to other national or regional language datasets for similar analyses (Supplementary material).

Briefly, we compiled a matrix of manually curated lexical items, with each row representing a language and each column corresponding to the same gloss. Missing entries were excluded pairwise during distance calculations to preserve alignment integrity. For each language pair, we computed LDN using the stringdist package in R. Specifically, for each pair of corresponding words, we calculated the Levenshtein (edit) distance and normalized it by the maximum string length to account for differences in word length. These normalized distances were then averaged across all word pairs, yielding a symmetric matrix of pairwise lexical distances. We visualized the resulting distance matrix using MDS coordinates in 2D and 3D scatterplots, with languages color-coded by clusters obtained from hierarchical agglomerative clustering (hclust, average linkage). These coordinates were used to generate scatterplots, with languages represented as points and labelled using ggrepel to reduce overlap, and visualized using ggplot2. Clustering results were further visualized using a radial dendrogram (via ape::plot.phylo). A heatmap was generated using pheatmap, and interactive 2D and 3D plots exported to HTML using plotly. These visualizations provide complementary perspectives on the internal structure of lexical similarity across language varieties. To assess the correlation between genetic distance (*F*_ST_) and lexical distance (LDN), we conducted pairwise Mantel correlation tests using the vegan package in R. The strength of correlation was evaluated using a correlation coefficient (r), where values approaching 1 indicate strong positive correlation.

This approach builds on evidence that there is a correlation between lexical and genetic differentiation, as demonstrated in comparative studies of phonemic, lexical, and genetic coevolution across global populations (8, 10).

## Results

**Figure 1:**
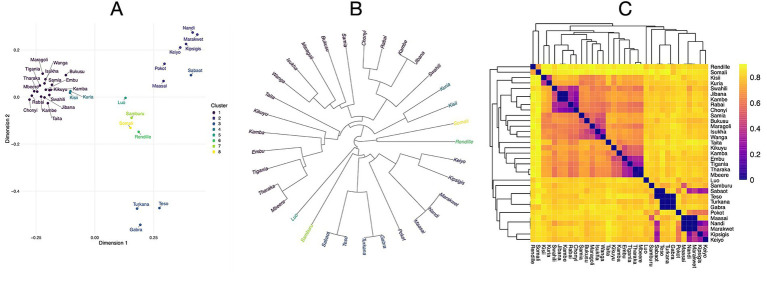
Lexical differentiation of Kenyan languages. **(A)** Multidimensional scaling (MDS) of normalized Levenshtein distances reveals distinct clustering of Kenyan ethnic groups according to their linguistic classifications within the Bantu, Nilotic, and Cushitic languages. **(B)** Dendrogram of hierarchical clustering analysis revealing distinct language family groupings. Bantu languages showing close relationships, Nilotic languages forming a moderately distant cluster with Kalenjin varieties as a notable sub-group, and Cushitic languages positioned as the most distant outgroup consistent with their Afro-Asiatic origins. **(C)** Heatmap visualization of pairwise lexical distances. There is strong association between lexical similarity and language family classification.

Lexical similarity analysis across language of different Kenyan ethnic groups revealed distinct clustering patterns. Multidimensional scaling (MDS) of normalized Levenshtein distances (LDN) produced a two-dimensional visualization that clearly separates ethnic groups from the three major language families: Bantu, Nilotic, and Cushitic (Figure 1A). This separation portrays differences associated with Kenya’s ethnolinguistic landscape that closely mirrors human migratory history into Kenya.

Hierarchical clustering analysis generated a dendrogram (Figure 1B) that further resolves the relationships within each of the three (Bantu, Nilotic, and Cushitic) major language groups. The Bantu cluster shows tight internal grouping with short branch lengths between languages such as Kikuyu, Kamba, and Luhya, indicating high lexical similarity consistent with their relatively recent divergence during the Bantu expansion approximately 2,000–3,000 years ago. The Nilotic languages form a distinct cluster with moderate internal distances, reflecting their shared ancestry but more ancient divergence patterns. Within this group, Kalenjin subcommunities (Kipsigis, Nandi) exhibit particularly close relationships, forming a distinct sub-cluster. The Cushitic languages appear as the most distant outgroup, consistent with their Afro-Asiatic origins and longer separation from the Niger-Congo and Nilo-Saharan language families.

Heatmap visualization of pairwise lexical distances (Figure 1C) reveals a clear block-like structure corresponding to the three major language families. Intra-family distances (diagonal blocks) show consistently lower values compared to inter-family distances (off-diagonal blocks), with the darkest blue regions indicating the closest lexical relationships. This pattern quantitatively confirms the strong association between lexical similarity and language family classification.

**Figure 2:**
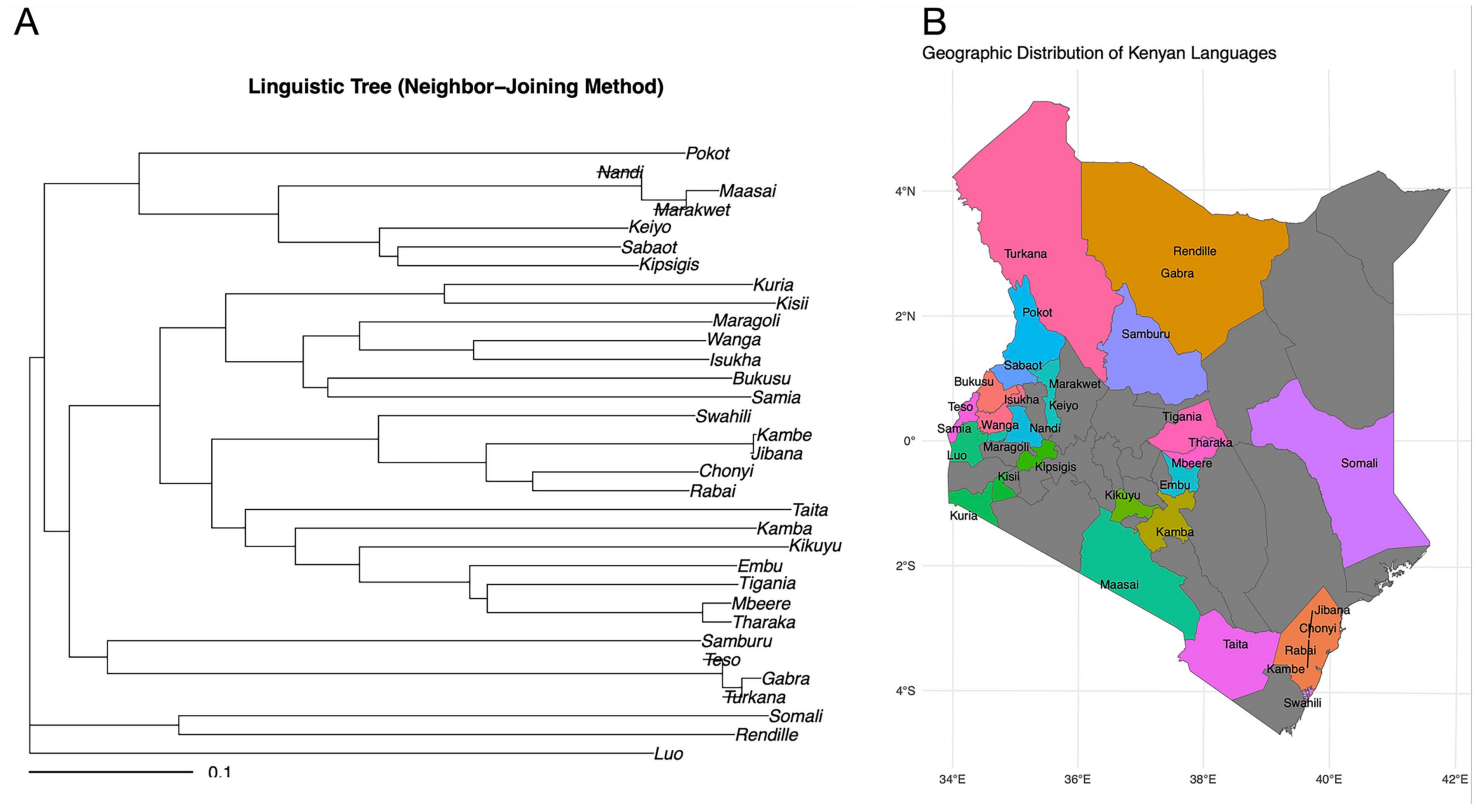
Comparison of lexical similarity to ethnic group geographic proximity. **(A)** Neighbour joining linguistic tree showing lexical (LDN) distance. **(B)** Geographic locations predominantly occupied represented ethnic groups. There are great lexical differences between Kikuyu (Bantu) and Maasai (Nilotic) ethnic groups despite close geographical proximity, compared to geographically separated Kikuyu and Luhya (both Bantu). The colors in the map show each language family used in the analysis, and the relative habitation location of community after migration into Kenya. The grey areas represent communities not represented in this study.

When comparing linguistic distance to geographic proximity, we found that comparisons between communities from different language families exhibit greater linguistic distance even when they live geographically adjacent to each other compared to more geographically distant communities from within the same language family (Figures 2A,B). For example, Kikuyu (Bantu) and Maasai (Nilotic) communities who were historically geographical neighbors maintain substantial lexical distance (LDN = 0.9), whereas the geographically separated Kikuyu and Luhya (both Bantu) show lower lexical distance (LDN = 0.67). This pattern is consistent across multiple language pairs (Figure 2A), with intra-family comparisons consistently showing lower LDN values than inter-family comparisons regardless of geographic proximity.

There is a paucity of human genetic data from Africa, making comparative analyses challenging. In Kenya, publicly accessible genetic population differentiation data is only available from the HapMap, 1,000 Genomes and African Genome Variation projects (2, 5, 13–15). We retrieved these available data and compared our lexical distance matrix with previously published genetic population differentiation fixation index (*F*_ST_) value data with overlapping populations (Luhya-vs-Kikuyu; Luhya-vs-Masaai; and Masaai-vs-Kikuyu) (Table 2) (19, 20). Mantel correlation test demonstrated a strong correlation between lexical and genetic differentiation [*r* = 0.91, CI (0.55–0.99), *p* = 0.09], which was notably stronger than the correlation between geographic and genetic differentiation [*r* = 0.29, CI (0.29–0.53), *p* = 0.001]. While the relationship between lexical distance and population differentiation showed a strong positive trend (*r* = 0.91), statistical significance was not reached due to paucity of data (Table 2).

**Table 2:** Population pairwise comparisons between population differentiation, and lexical distance.

Population pair	Population differentiation (*F*_ST_ values)	Lexical distance (LDN values)
Luhya vs. Kikuyu	0.01	0.67
Kikuyu vs. Maasai	0.1	0.85
Luhya vs. Maasai	0.17	0.82
Maasai vs. Kalenjin	0.06	0.8

**Figure 3:**
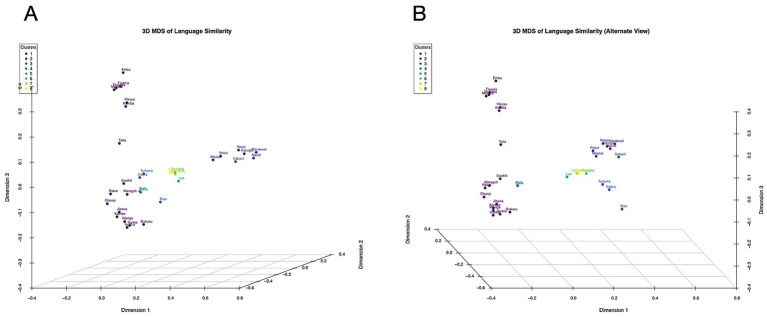
The three-dimensional MDS plot reveals subtle deviations from strict phylogenetic clustering. Languages at geographic interfaces, for example Swahili and Teso, show intermediate positioning.

We further examined how borrowing between neighbouring languages affects lexical similarity patterns. The three-dimensional MDS plot (Figure 3) reveals subtle deviations from strict phylogenetic clustering, with languages at geographic interfaces showing intermediate positioning. For instance, Swahili (Bantu) appears slightly displaced toward Cushitic languages, consistent with its documented lexical borrowing from Arabic and Somali through centuries of coastal trade interactions (21).

Our analysis identified several key “bridge populations” that exhibit mixed lexical influences from multiple language families, particularly Teso and Turkana (Figure 3), which show Nilotic classification but position between Nilotic and Cushitic clusters in multidimensional space. This suggests historical interactions between these pastoralist communities and neighbouring Cushitic groups in northern Kenya.

This supports reports on divergence of genes and languages due to language replacement, interactions across significant geographical distances, often involving trade and migration, and horizontal cultural transmission (10, 11).

Our sampling strategy provides an objective and replicable quantitative basis for prioritizing populations for genomic studies that maximizes genetic diversity represented, while minimizing redundancy and saving costs (22–24).

## Discussion

We suggest that lexical analysis can be used as a proxy to prioritise multi-ethnic ancestries for populations where human genetic data is limited. Language evolution closely mirrors demographic history for example migration, admixture, and isolation (5, 15). Lexical similarity suggests shared ancestry, sustained interaction between populations, historical contact, or recent divergence. This quantitative analytical framework offers finer resolution through continuous measures of lexical similarity rather than categorical language classifications.

Clear separation of Kenya’s three major language families (Bantu, Nilotic, and Cushitic) in lexical space mirrors their distinct migration histories and origins, consistent with previous reports of human movement in Eastern Africa (16, 17). Our analysis demonstrates that lexical similarity patterns effectively predict genetic relatedness among Kenyan populations, providing a powerful framework for prioritizing population sampling in genomic studies. The strong correlation between lexical and genetic population differentiation (*r* = 0.91) confirms that shared linguistic heritage closely mirrors genetic ancestry in the multi-ethnic context of Africa. A recent genome-wide study of populations in the Horn of Africa (HOA), to understand human migration patterns, found no significant correlation between genetic and geographic distance when compared to neighbouring populations Middle-East and North Africa (MENA) (25). By contrast, analysis of molecular variance (AMOVA) revealed significant genetic differentiation among linguistic groups within the HOA populations highlighting the utility of integrating of lexical classifications alongside genetic data to better capture population structure and diversity (25). This supports observation that the Y chromosome shows a strong relationship with language groups regardless of geography, suggesting patrilocal practices where males tend to remain in their linguistic communities (7). This implies that cultural and linguistic boundaries have maintained strong barriers to gene flow than geographic distance alone, even between adjacent ethnic populations. We show that language serves as a better proxy for population history than geography across Africa, and provide quantitative lexical distance methodology that enables systematic prioritization of populations for genomic sampling.

The identification of “bridge populations” with lexical features intermediate between major language groups highlights the complexity of vertical inheritance and horizontal transfer in both linguistic and genetic evolution (26). These populations, particularly those at the interface of different language families, may represent important targets for genomic studies seeking to understand admixture processes and recent population history in Africa (27). We distinguish differentiated populations despite geographic proximity and previous cultural contact, reflecting deep population history despite recent interactions. The optimized sampling strategy derived from our lexical framework provides an avenue for genomic researchers seeking to capture human genetic diversity in understudied populations. Prioritizing representatives from distinct lexical clusters can help address the significant underrepresentation of African genetic diversity in global databases while making efficient use of limited sequencing resources. This approach provides an objective method for sampling strategy development that moves beyond convenience sampling often used in human genetic studies (28). Reducing redundancy in sequencing efforts among underrepresented populations with predominant African-related genetic similarity will enable more strategic and efficient sampling designs. Bridge populations prioritized for sequencing highlight admixture history and recent population dynamics, providing unique insights into human adaptation and demographic history that would be missed by previous sampling strategies. This method provides an avenue to increase access to genomic data from underrepresented populations and is generalizable across diverse ancestral backgrounds. A recent article underscores the importance of adopting a pangenomic approach to enhance population genetics characterization analyses and reduce reference bias associated with the hg38 genome, particularly in underrepresented populations (29). Although this state-of-the-art, graph-based approach offers improved robustness, it is computationally intensive and requires substantial resources. However, it is hoped that future support for this work will enable this issue to be addressed in later studies.

A limitation in our study was the paucity of human genetic data (Luhya, Maasai and Kikuyu pair-wise genetic population distance data) to conduct comparative analyses between lexical and genetic differentiation, highlighting the need for novel population prioritization and sampling strategies.

In conclusion, this lexical similarity analysis framework could provide a roadmap for more inclusive and strategic genetic research in populations with predominant African-related genetic similarity, potentially accelerating efforts to address the significant underrepresentation in global genetic data catalogues.

## Data Availability

The original contributions presented in the study are included in the article/Supplementary material, further inquiries can be directed to the corresponding author/s.
