# Supplementary material for: Operationalizing language-based population stratification for widening access to precision genomics in Africa
Source: Front Public Health. 2025 Sep 12;13:1672038. doi: 10.3389/fpubh.2025.1672038 (PMC12463837; doi:10.3389/fpubh.2025.1672038)
Supplement: Supplementary file 1 [file Table_1.DOCX]

| **1. Bantu Languages** |
| --- |
| - Kikuyu (Gikuyu) |
| - Kamba |
| - Embu |
| - Mbeere |
| - Tigania |
| - Tharaka |
| - Kisii |
| - Kuria |
| - Swahili |
| - Taita |
| - Mijikenda languages: |
| - - Jibana |
| - - Rabai |
| - - Kambe |
| - - Chonyi |
| - Luhya dialects: |
| - - Isukha |
| - - Wanga |
| - - Samia |
| - - Bukusu |
| - - Maragoli |
| **2. Nilotic Languages** |
| - Luo (Dholuo) |
| - Kalenjin languages: |
| - - Kipsigis |
| - - Nandi |
| - - Keiyo |
| - - Marakwet |
| - - Sabaot |
| - - Pokot |
| - Maasai |
| - Turkana |
| - Samburu |
| - Teso |
| **3. Cushitic Languages** |
| - Rendille |
| - Somali |
| - Gabra |
