# Supplementary material for: Operationalizing language-based population stratification for widening access to precision genomics in Africa
Source: Front Public Health. 2025 Sep 12;13:1672038. doi: 10.3389/fpubh.2025.1672038 (PMC12463837; doi:10.3389/fpubh.2025.1672038)
Supplement: Supplementary file 2 [file Data_Sheet_1.ZIP › interactive_3D_MDS.html]

---
title: plotly
header-include: |


head: |2+
background-color: white
---
