# Supplementary material for: Operationalizing language-based population stratification for widening access to precision genomics in Africa
Source: Front Public Health. 2025 Sep 12;13:1672038. doi: 10.3389/fpubh.2025.1672038 (PMC12463837; doi:10.3389/fpubh.2025.1672038)
Supplement: Supplementary file 3 [file Data_Sheet_2.ZIP › Supplementary_materials_v2/Dataset_&_script_2.zip!/Dataset_&_script_2/linguistic_tree.pdf]

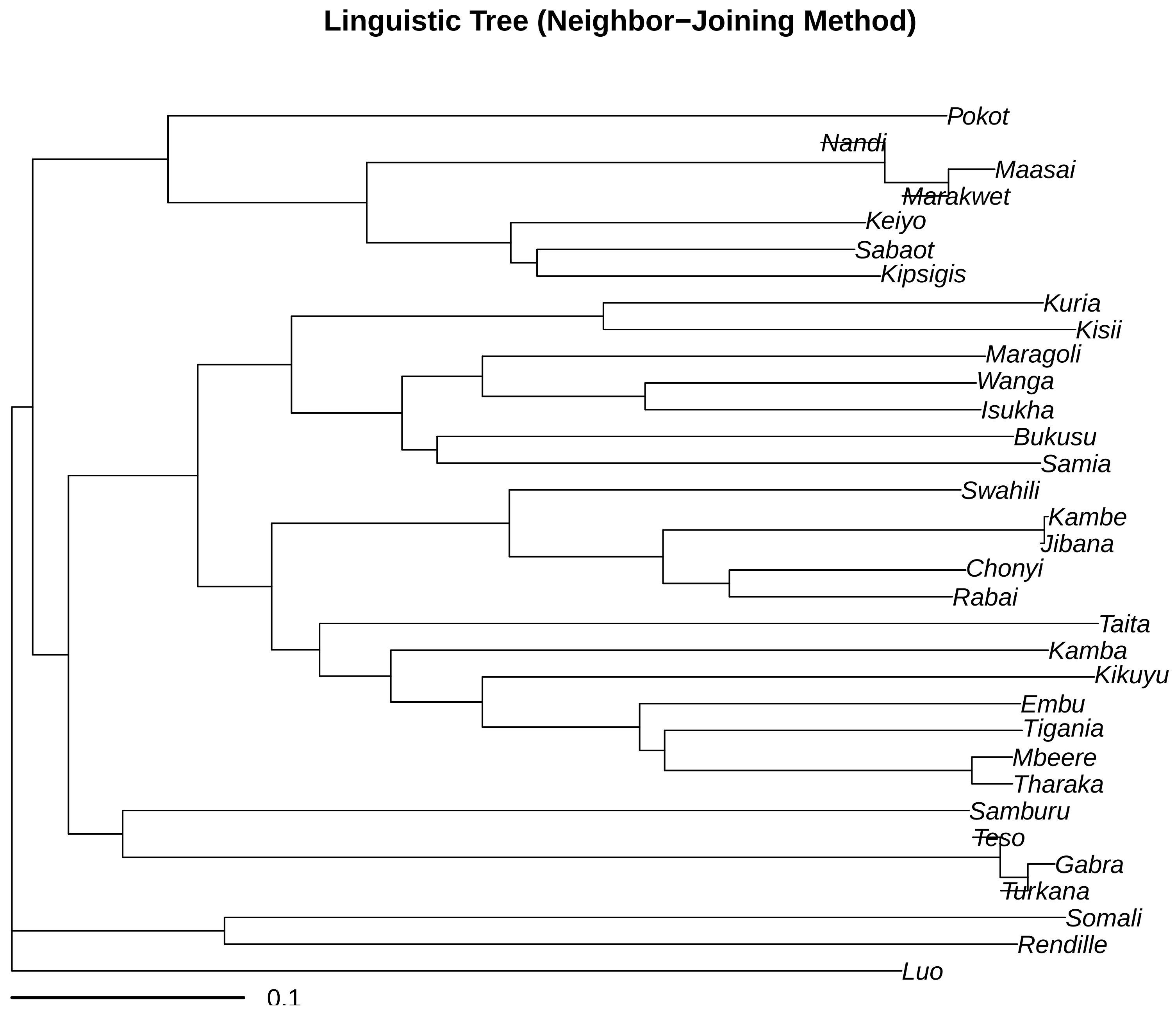

Linguistic Tree (Neighbor−Joining Method)
Pokot
Nandi
Maasai
Marakwet
Keiyo
Sabaot
Kipsigis
Kuria
Kisii
Maragoli
Wanga
Isukha
Bukusu
Samia
Swahili
Kambe
Jibana
Chonyi
Rabai
Taita
Kamba
Kikuyu
Embu
Tigania
Mbeere
Tharaka
Samburu
Teso
Gabra
Turkana
Somali
Rendille
Luo
0.1
