# Supplementary material for: Operationalizing language-based population stratification for widening access to precision genomics in Africa
Source: Front Public Health. 2025 Sep 12;13:1672038. doi: 10.3389/fpubh.2025.1672038 (PMC12463837; doi:10.3389/fpubh.2025.1672038)

Geographic Distribution of Kenyan Languages

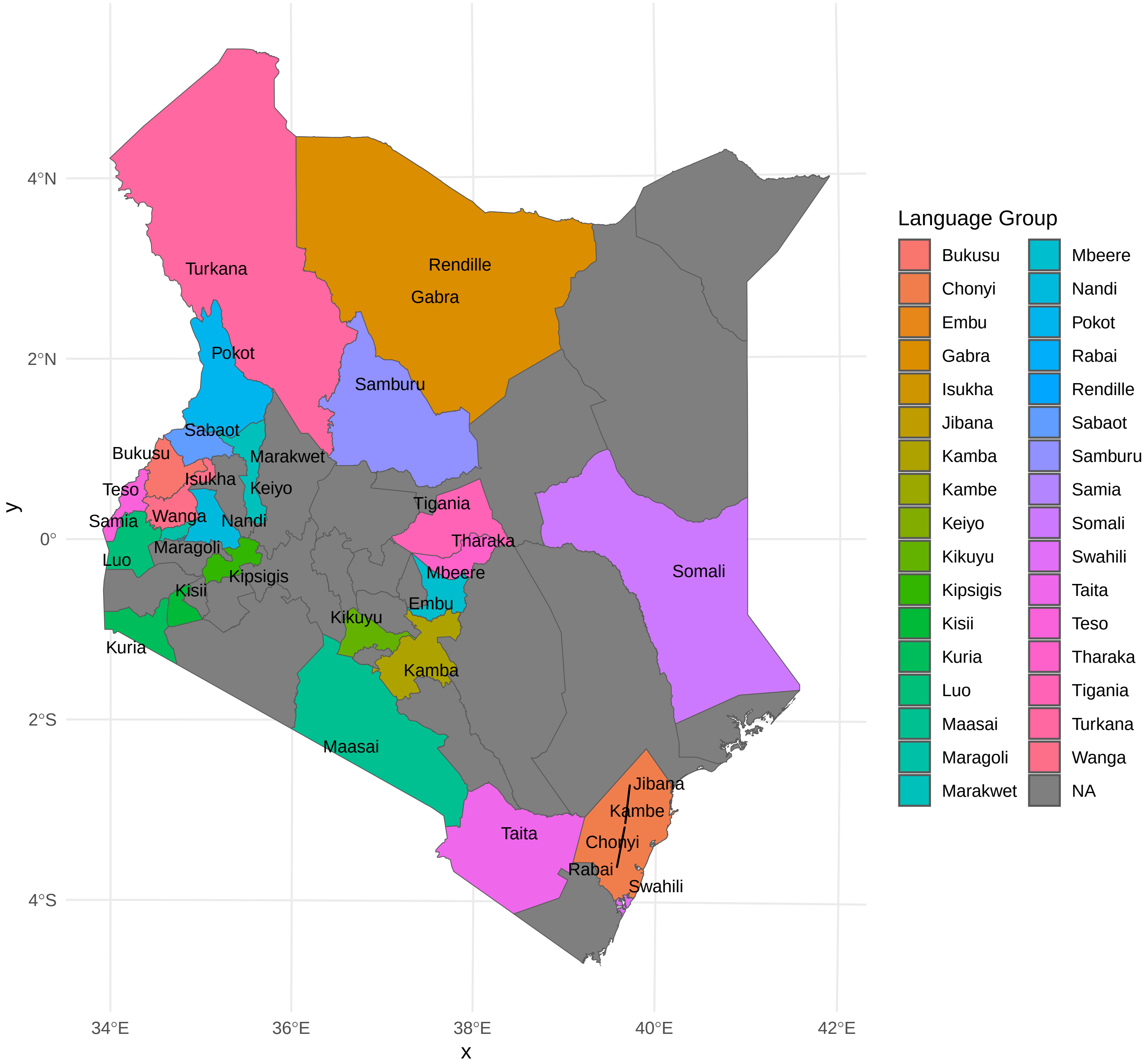

Supplement: Supplementary file 3 [file Data_Sheet_2.ZIP › Supplementary_materials_v2/Dataset_&_script_2.zip!/Dataset_&_script_2/geographic_distribution.pdf]
